# A biological Indian Ocean Dipole event in 2019

**DOI:** 10.1038/s41598-021-81410-5

**Published:** 2021-01-28

**Authors:** Wei Shi, Menghua Wang

**Affiliations:** 1 NOAA National Environmental Satellite, Data, and Information Service, Center for Satellite Applications and Research, E/RA3, 5830 University Research Ct., College Park, MD 20740 USA; 2 CIRA at Colorado State University, Fort Collins, CO 80523 USA

**Keywords:** Marine biology, Physical oceanography

## Abstract

The 2019 positive Indian Ocean Dipole (IOD) event in the boreal autumn was the most serious IOD event of the century with reports of significant sea surface temperature (SST) changes in the east and west equatorial Indian Ocean. Observations of the Visible Infrared Imaging Radiometer Suite (VIIRS) onboard the Suomi National Polar-orbiting Partnership (SNPP) between 2012 and 2020 are used to study the significant biological dipole response that occurred in the equatorial Indian Ocean following the 2019 positive IOD event. For the first time, we propose, identify, characterize, and quantify the biological IOD. The 2019 positive IOD event led to anomalous biological activity in both the east IOD zone and west IOD zone. The average chlorophyll-a (Chl-a) concentration reached over ~ 0.5 mg m^−3^ in 2019 in comparison to the climatology Chl-a of ~ 0.3 mg m^−3^ in the east IOD zone. In the west IOD zone, the biological activity was significantly depressed. The depressed Chl-a lasted until May 2020. The anomalous ocean biological activity in the east IOD zone was attributed to the advection of the higher-nutrient surface water due to enhanced upwelling. On the other hand, the dampened ocean biological activity in the west IOD zone was attributed to the stronger convergence of the surface waters than that in a normal year.

## Introduction

The Indian Ocean Dipole (IOD) is an ocean–atmosphere phenomenon in the tropical Indian Ocean^1–3^. As a measure of the IOD, Dipole Mode Index (DMI), which is calculated as the difference of the sea surface temperature (SST) anomaly in the eastern equatorial Indian Ocean (90°E–110°E, 10°S–0°N) and the SST anomaly in the western equatorial Indian Ocean (50°E–70°E, 10°S–10°N)^1^, measures the development and the strength of the IOD. During the positive IOD event, the stronger-than-normal southeasterly winds along the Sumatra coast cause stronger coastal upwelling and SST cooling^4,5^. The excited upwelling Kelvin wave reflects from the eastern coast and propagates westward as the upwelling Rossby waves^5^. The eastern equatorial Indian Ocean featured cold SST anomalies (SSTA), lower sea level, and shallow thermocline to the south of the equator. The western pole of the IOD is marked by warm SSTA, and the thermocline becomes deeper with maxima on the either side of the equator^6^. Some of the IOD events can be linked to and interact with tropical Pacific variabilities including El Niño Southern Oscillation (ENSO)^7–9^. Recent studies show that the IOD events can be predicted a couple of seasons before the IOD event occurs^8,10^.

As a basin-wide ocean–atmosphere phenomenon, the IOD exerts impacts on global climate^11^. Indeed, the IOD has a significant influence on the Indian summer monsoon^12^. The anomalous rainfall in the East Africa^13^, South America^14^, and South Asia^15^ due to the IOD event was reported. The most severe drought in a large part of the Australia was also linked the IOD^16^ as its main driving force. In fact, the positive IOD events were preconditions for the southeast Australia bushfire^17^. Eleven significant bushfire seasons since 1950 were related to positive IOD events due to dry conditions and high air temperature. Wild fires also contributed to coral reef bleaching during the 1997 IOD event^18^. Natural hazards such as the occurrence of locust plagues and outbreaks of malaria in Africa are also related to the climate variability caused by the IOD^19,20^.

In the 2006 positive IOD event, anomalous negative subsurface temperature at the thermocline depth was observed to move westward three months before the IOD event was identified^21^. The IOD strongly modulated the upper ocean variability in the tropical Indian Ocean^22^. The subsurface variability was governed and characterized by the IOD in the tropical Indian Ocean^23^. In addition, the IOD events also have significant impacts on the regional biological activities in the Indian Ocean^24^. Phytoplankton bloom was observed at the coastal regions of Sumatra and Java in the 2006 IOD event^25^. In the northwestern Bay of Bengal, the IOD also drove the phytoplankton bloom^26^. Indeed, an unprecedented phytoplankton bloom in the southeastern Arabian Sea was reported during the extreme negative IOD event in 2016^27^. Furthermore, the IOD is also found to influence the variation of phytoplankton size structure^28^.

In this study, Chl-a concentration, a surrogate for the ocean biological activity, is quantified and analyzed in the east and west IOD regions from observations of the Visible Infrared Imaging Radiometer Suite (VIIRS) onboard the Suomi National Polar-Orbiting Partnership (SNPP) satellite since 2012. In combination with physical driving forces such as the wind variability and vertical velocity in the ocean subsurface layer, the mechanism that drove nutrient dynamics and thus caused the anomalous biological activity is further addressed and explored.

## Data and methods

### VIIRS-SNPP-derived Chl-a product

As the follow-on Earth observation mission from the Moderate Resolution Imaging Spectroradiometer (MODIS) onboard the Terra and Aqua satellites, VIIRS-SNPP has delivered continuous operational satellite data streams for global atmosphere, land, cryosphere, and ocean/water products since 2012. The normalized water-leaving radiance *nL*_*w*_(*λ*) spectra, which are the key to produce high-quality satellite ocean color products, are vicariously calibrated on-orbit with the in-situ *nL*_*w*_(*λ*) measurements at the Marine Optical Buoy (MOBY)^29,30^ site off the Hawaii Island. The VIIRS-SNPP ocean color products are generated with the Multi-Sensor Level-1 to Level-2 (MSL12) ocean color data processing system at NOAA^31^. Specifically, Chl-a products are produced using the ocean color index (OCI) Chl-a algorithms for the global ocean^32–35^.

In this study, the monthly composites of Chl-a data are used to investigate the temporal variations of Chl-a and the Chl-a anomaly during the 2019–2020 IOD event in the equatorial Indian Ocean. The monthly climatology Chl-a were computed from all Chl-a data since 2012 as a reference for the Chl-a temporal variability, and were used to further quantify and characterize the Chl-a anomaly during the 2019 IOD event. Corresponding to the SSTA in the west pole region (50°E–70°E, 10°S–10°N) and east pole region (90°E–110°E, 10°S–0°N) that defines the DMI, the Chl-a dynamics and Chl-a anomalies in these two regions are also quantified and characterized in order to demonstrate the biological IOD in the region.

### DMI, SST, wind fields, and subsurface vertical velocity

To understand the physical and nutrient dynamics that drove the biological IOD, we also examined the DMI data, wind velocity, SST, and the subsurface vertical velocity. All of these datasets were acquired from the NOAA Physical Sciences Laboratory (https://psl.noaa.gov/).

The DMI data are a monthly SSTA difference between western equatorial Indian Ocean (50°E–70°E and 10°S–10°N) and the southeastern equatorial Indian Ocean (90°E–110°E and 10°S–0°N)^1^. The NOAA high-resolution blended SST data^36^ have a spatial resolution of 1/4°. It is produced by combining measurements from various platforms such as satellites, ships, buoys, etc., in each grid.

National Centers for Environmental Prediction (NCEP)/National Center for Atmospheric Research (NCAR) reanalysis monthly surface wind data^37^ have a spatial resolution of 2.5°. The analysis/forecast system is used to conduct the data assimilation using data since 1948 with 4-time/day, daily, and monthly temporal resolutions. The subsurface vertical velocity data are the output of the NCEP Global Ocean Data Assimilation System (GODAS)^38,39^. GODAS is a real-time ocean analysis and reanalysis system. Both the ocean temperature and salinity profiles are assimilated in a three-dimensional variational data assimilation (3DVAR) scheme. There are 40 vertical levels in the model. The data are provided at 1/3 × 1/3 degrees for all latitudes.

Corresponding to the observation period of VIIRS Chl-a since 2012, the monthly means of the wind, SST, and vertical velocity data from 2012 to 2019 are calculated as the monthly climatology wind, monthly climatology SST, and monthly climatology vertical velocity. The monthly climatology wind field, SST, and vertical velocity are used as the references to characterize and quantify the anomalies of these physical parameters in order to identify the driving mechanism for the biological IOD in the 2019 IOD event.

## Results

### The 2019 IOD event

The equatorial Indian Ocean experienced the strongest positive IOD of the century and the DMI reached the same level of the IOD event back in 1997^8^. This extreme positive IOD event started with the anomalous inter-hemisphere pressure gradient (IHPG) between the strengthening pressure over Australia and weakening pressure over the region of the South China Sea/Philippine Sea^40^. In this IOD event, the air-sea heat flux further enhanced the cold SST in the eastern equatorial Indian Ocean instead of dampening the cold SST in the other positive IOD events^41^. It is also found that the thermocline warming in the southwestern tropical Indian Ocean also contributed to the development of this IOD event^42^.

**Figure 1:**
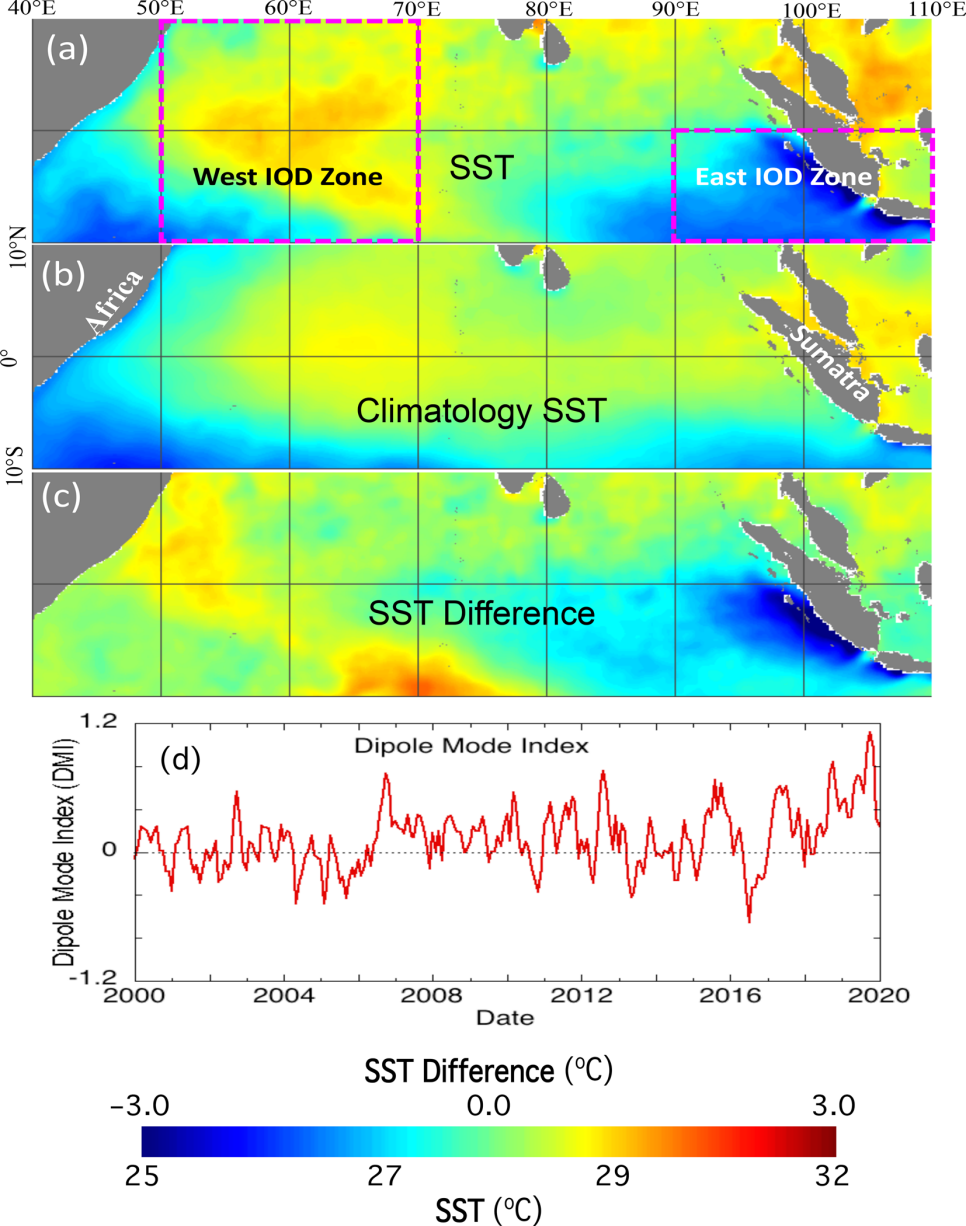
(**a**) SST in October 2019 in the equatorial Indian Ocean, (**b**) mean climatology SST in the equatorial Indian Ocean between 2012 and 2019, (**c**) SST difference between SST in October 2019 and the same-month climatology SST, (**d**) temporal variation of the DMI since 2000. Note that the west IOD zone (50°E‒70°E, 10°S‒10°N) and the east IOD zone (90°E‒110°E, 10°S‒0°N) are marked in Fig. 1a.

Figure 1a shows SST in the equatorial Indian Ocean in October 2019, while Fig. 1b is the climatology SST in the same month. Indeed, the southeastern equatorial Indian Ocean exhibited low SST in the region of 90°E‒110°E. On the other hand, SST in the western equatorial Indian Ocean in October 2019 was remarkably higher than the climatology SST in the same month.

The SST difference between the SST in October 2019 and the climatology SST reached over − 3 °C near the Sumatra coastal region (Fig. 1c). For most of the east IOD zone, the SST difference was in the range of − 3 to − 1 °C. In the west IOD zone, SST in October 2019 was about 1–2 °C higher than the climatology SST.

The DMI time series (Fig. 1d) indeed shows that the IOD in the 2019 boreal autumn was the strongest one in the last 20 years. The DMI values in September, October, and November of 2019 were 0.999, 1.123, and 0.958, respectively. The DMI dropped to ~ 0.312 in December 2019. In comparison, the positive DMI was only 0.736 in October 2006 in another strong IOD event, which led to a significant response in the equatorial Indian Ocean^6,25^.

### Enhanced Chl-a anomaly in the east IOD zone

**Figure 2:**
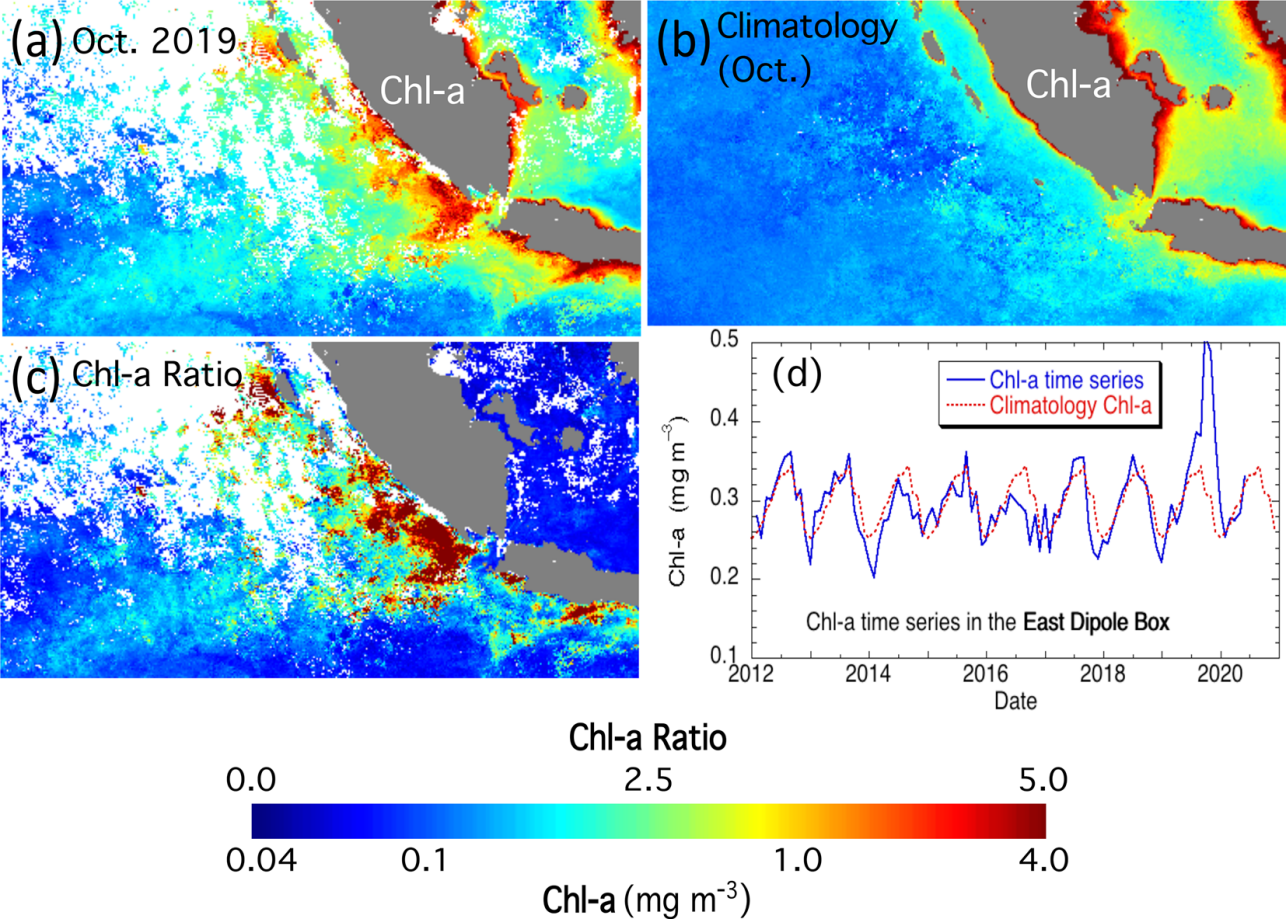
(**a**) Chl-a in October 2019, (**b**) Chl-a climatology in October, and (**c**) the ratio of Chl-a in October 2019 and climatology Chl-a in October in the east IOD zone. (**d**) Time series of the average Chl-a between 2012 and mid-2020 in the east IOD zone. The seasonal climatology average Chl-a is shown as a dashed line for comparison.

Chl-a in the east IOD zone were broadly enhanced due to a phytoplankton bloom (Fig. 2a). In the Sumatra coastal region, Chl-a reached ~ 3 to 4 mg m^−3^ in October 2019 in comparison to the typical value of < 1 mg m^−3^ in the climatology Chl-a in the same month (Fig. 2b). In the region to the west of Sumatra and Java, Chl-a were observed to increase significantly. On the other hand, Chl-a in the region to the east of Sumatra and Java were similar to those in the climatology Chl-a.

The map of the Chl-a ratio for October 2019 and October climatology clearly shows the phytoplankton bloom in the east IOD zone (Fig. 2c). In the west Sumatra coastal region, the Chl-a ratio reached ~ 4 to 5, while the Chl-a ratio was over ~ 2 for most of the east IOD zone to the west of Sumatra and Java. The temporal variation in the monthly Chl-a in the east IOD zone further highlights the anomalous phytoplankton bloom in autumn 2019 (Fig. 2d). The monthly climatology Ch-a in this region ranged between ~ 0.25 and ~ 0.35 mg m^−3^ in a year. In the period between 2012 and mid-2019, the monthly Chl-a were more or less following the seasonal climatology Ch-a variation. However, Chl-a spiked in the boreal autumn of 2019. Indeed, the monthly Ch-a were ~ 0.50 and ~ 0.49 mg m^−3^ in October and November of 2019, respectively. As a comparison, the climatology monthly Chl-a were both ~ 0.31 mg m^−3^ in the same two months. In early 2020, however, Ch-a in this region were back to normal.

### Depressed Chl-a anomaly in the west IOD zone

**Figure 3:**
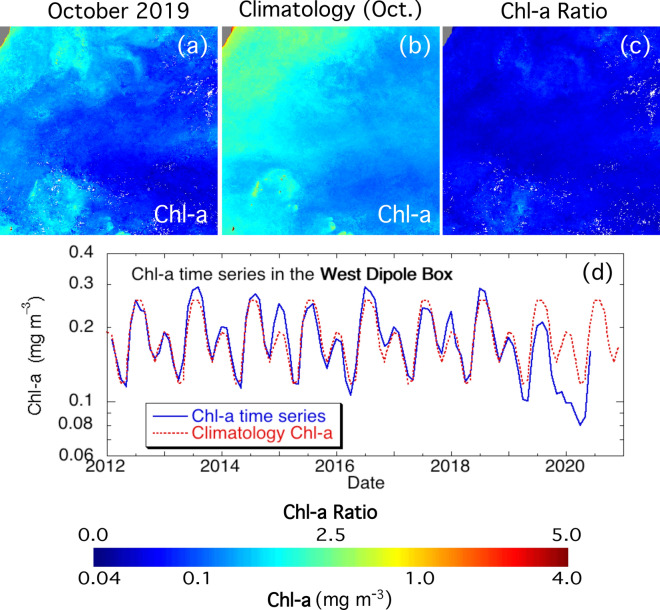
(**a**) Chl-a in October 2019, (**b**) Chl-a climatology in October, and (**c**) the ratio of Chl-a in October 2019 and climatology Chl-a in October in the west IOD zone. (**d**) Time series of the average Chl-a between 2012 and mid-2020 in the west IOD zone. The seasonal climatology average Chl-a is shown as a dashed line for comparison.

Similar to the east IOD zone, Chl-a in the west IOD zone during the 2019 IOD event was also examined and compared to the seasonal climatology Chl-a from VIIRS observations. Contrary to the east IOD zone, monthly Chl-a in October 2019 (Fig. 3a) were significantly lower than the climatology Chl-a in the same month (Fig. 3b) for both the coastal and offshore regions. To the west of the IOD zone, enhanced Chl-a over ~ 0.3 mg m^−3^ disappeared in October 2019. The Chl-a ratio in this month (Fig. 3c) shows that Chl-a in October 2019 was depressed to about 50%‒80% of Chl-a data in a normal year in the region.

Time series of Chl-a in this region also showed the anomalous biological activity during the 2019 IOD event (Fig. 3d). In this region, the seasonal change of Chl-a is significant from the monthly Chl-a climatology. The highest Chl-a value occurs in July and August at ~ 0.26 mg m^−3^ and the lowest occurs in April at ~ 0.12 mg m^−3^. In January and February, Chl-a reach a mini-peak at ~ 0.19 mg m^−3^. Before the 2019 IOD event, the seasonal change in the west IOD zone followed the climatology Chl-a with much less variations. Chl-a were actually shown to be remarkably less than the climatology Chl-a in May and June 2019 before the onset of the IOD event. The depressed Chl-a in this region lasted until April and May of 2020. In comparison to Chl-a in the other years, the mini-peak of Chl-a in early 2020 did not show up, while Chl-a were only about half of the climatology Chl-a during these two months.

### Driving force for the biological IOD

**Figure 4:**
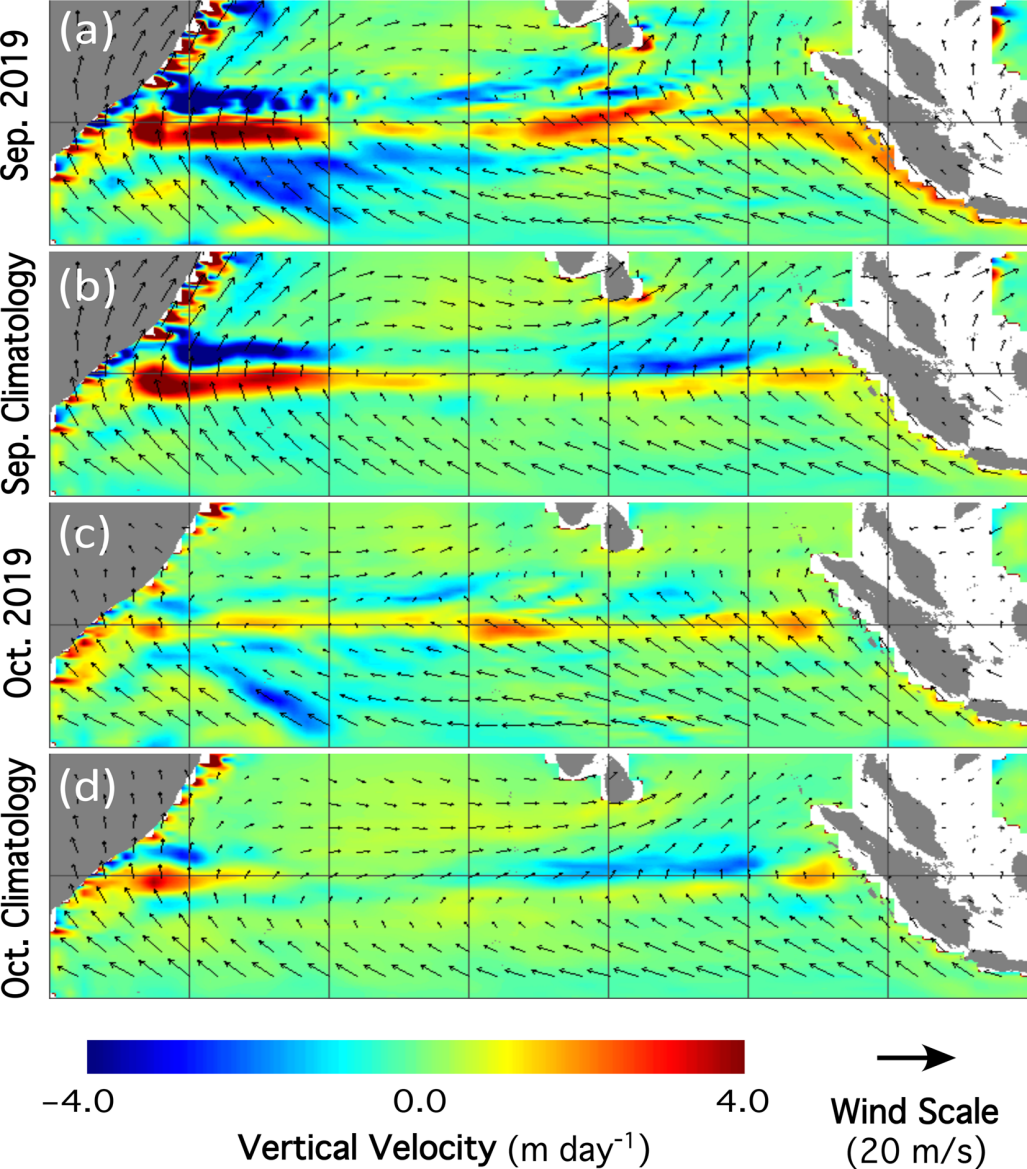
Comparisons of vertical velocity at 50 m depth with overlay of the surface wind velocity for (**a**) vertical velocity in September 2019, (**b**) climatology vertical velocity in September, (**c**) vertical velocity in October 2019, and (**d**) climatology vertical velocity in October.

As an ocean–atmosphere event, the ocean dynamics of the IOD event is closely related to the atmospheric processes. In September and October of 2019, the upwelling-favorable southeasterly winds along the Sumatra west coast was about 20–30% stronger than the climatology winds as shown in Fig. 4a‒d. In the east equatorial Indian Ocean near the equator, the easterly winds were also stronger than the climatology winds in September and October. These were consistent with the driving force and precondition for the positive IOD^4,5^.

In the equatorial Indian Ocean, the surface mixed layer depth is normally at the range of 30–60 m. Thus, the vertical speed at the depth of 50 m is well representative for the Ekman pumping in the thermocline depth. In September 2019, the wind anomaly along the Sumatra coast and the east equatorial Indian Ocean led to the enhanced upwelling in the east IOD zone (Fig. 4a). The upwelling speed along the Sumatra coast could reach over ~ 3 m/day, while it is normally < 1 m/day in a normal year (Fig. 4b). In October 2019, the enhanced upwelling faded (Fig. 4c), but it was still stronger than the climatology monthly vertical velocity in October (Fig. 4d).

In the west IOD zone, more downwelling was shown in September 2019 (Fig. 4a) than the climatology vertical velocity at 50 m depth (Fig. 4b). This is especially true in the southern part of the west IOD zone. The downwelling velocity was ~ 2 m/day in September 2019, while the climatology monthly vertical velocity in September was near 0 (Fig. 4b). In October 2019, anomalous downwelling in the west IOD region still existed (Fig. 4c, d) even though the downwelling was not as significant as that in September 2019.

Furthermore, the negative SST anomaly in the eastern IOD zone could be attributed to the advections of the upwelled low temperature waters due to the surface current and propagations of the equatorial Rossby waves. It is also noted that the highest SST anomaly occurred in October, and lagged the maximum upwelling anomaly in September 2019. Similarly, the surface water convergence and the deepening thermocline due to the stronger downwelling at the thermocline depth also drove the positive SST anomaly in the west IOD zone.

Nutrients such as nitrite and phosphate are critical for algal growth and phytoplankton bloom. In the equatorial Indian Ocean, the nutrient concentrations increase significantly with the increase of water depth^43^. As an example, the nitrite concentration increases from ~ 0.5 μmol/kg in the surface to ~ 1.6 μmol/kg at the depth of 50 m in the central east IOD zone. The nitrate concentration could reach > 10 μmol/kg at the bottom of the thermocline. This implies that the enhanced upwelling driven by the anomalous winds in autumn 2019 brought up the high-nutrient sub-surface water to the surface mixed layer, and the offshore advection of the high-nutrient surface led to the phytoplankton bloom in the east IOD region.

In the west IOD zone, however, the enhanced downwelling in the subsurface layer in September and October 2019 suggests the convergence of the surface waters and the deepening of the thermocline in the 2019 IOD event. This could lead to the further deficiency of nutrients for the phytoplankton growth, and consequently caused sustained low Chl-a anomaly in the west IOD zone. It is also noted that the low Chl-a anomaly in this region lasted well into May 2020 as shown in Fig. 3d, while the SST anomaly in the region was already back to normal in early 2020.

It should be also noted that the biological IOD reversed in the 2016 negative IOD event. Figure 1d shows that negative IOD occurred in 2016 with DMI dropping below − 0.5 °C. In that year, Chl-a in the east IOD zone were notably less than the climatology Ch-a (Fig. 2d). This suggests that dampening biological activities happened during the negative IOD event in the region. Contrary to the 2019 positive IOD, Chl-a in the west IOD zone were slightly enhanced in 2016 in comparison to the climatology Chl-a (Fig. 3d). Examinations of the vertical velocity in 2016 also show that indeed there was the opposite ocean dynamics such as weakening upwelling in the east IOD zone during the 2016 negative IOD event.

## Conclusion

In this study, VIIR-SNPP observations show significant biological changes in the equatorial Indian Ocean following the 2019 IOD event. Using the east and west IOD zones as defined for the DMI, we show the biological IOD in the equatorial Indian Ocean during the 2019 positive IOD event. Chl-a in the east IOD zone increased to over ~ 0.5 mg m^−3^ in October 2019 from the climatology (normal) Chl-a of ~ 0.3 mg m^−3^. In the west IOD zone, Chl-a dropped more than 30% in the 2019 IOD event in comparison to the climatology Chl-a, and the depressed Chl-a in the west IOD zone lasted until May 2020.

Analysis of the driving force in the 2019 IOD event shows that stronger wind along the Sumatra coast and east equatorial Indian Ocean in 2019 led to enhanced upwelling. The enhanced upwelling can bring high-nutrient subsurface water to the surface layer. The phytoplankton bloom during the 2019 IOD event is attributed to higher nutrient supplies in the east IOD zone following the advection of the high-nutrient upwelling water in the region. On the other hand, the weaker Chl-a in the west IOD zone is attributed to the nutrient deficiency due to the convergence of the surface water and the thermocline deepening in the west equatorial Indian Ocean.
